# Dietary vitamin D3 deficiency exacerbates sinonasal inflammation and alters local 25(OH)D_3_ metabolism

**DOI:** 10.1371/journal.pone.0186374

**Published:** 2017-10-18

**Authors:** Jennifer K. Mulligan, Whitney N. Pasquini, William W. Carroll, Tucker Williamson, Nicholas Reaves, Kunal J. Patel, Elliott Mappus, Rodney J. Schlosser, Carl Atkinson

**Affiliations:** 1 Department of Otolaryngology-Head & Neck Surgery, Medical University of South Carolina, Charleston, South Carolina, United States of America; 2 Department of Pediatrics, Medical University of South Carolina, Charleston, South Carolina, United States of America; 3 Department of Microbiology & Immunology, Medical University of South Carolina, Charleston, South Carolina, United States of America; 4 Department of Surgery, Lee Patterson Allen Transplant Immunobiology Laboratory, Medical University of South Carolina, Charleston, South Carolina, United States of America; 5 Ralph H. Johnson VA Medical Center, Charleston, South Carolina, United States of America; Telethon Institute for Child Health Research, AUSTRALIA

## Abstract

**Rationale:**

Patients with chronic rhinosinusitis with nasal polyps (CRSwNP) have been shown to be vitamin D3 (VD3) deficient, which is associated with more severe disease and increased polyp size. To gain mechanistic insights into these observational studies, we examined the impact of VD3 deficiency on inflammation and VD3 metabolism in an *Aspergillus fumigatus* (Af) mouse model of chronic rhinosinusitis (Af-CRS).

**Methods:**

Balb/c mice were fed control or VD3 deficient diet for 4 weeks. Mice were then sensitized with intraperitoneal Af, and one week later given Af intranasally every three days for four weeks while being maintained on control or VD3 deficient diet. Airway function, sinonasal immune cell infiltrate and sinonasal VD3 metabolism profiles were then examined.

**Results:**

Mice with VD3 deficiency had increased Penh and sRaw values as compared to controls as well as exacerbated changes in sRaw when coupled with Af-CRS. As compared to controls, VD3 deficient and Af-CRS mice had reduced sinonasal 1α-hydroxylase and the active VD3 metabolite, 1,25(OH)_2_D_3_. Differential analysis of nasal lavage samples showed that VD3 deficiency alone and in combination with Af-CRS profoundly upregulated eosinophil, neutrophil and lymphocyte numbers. VD3 deficiency exacerbated increases in monocyte-derived dendritic cell (DC) associated with Af-CRS. Conversely, T-regulatory cells were decreased in both Af-CRS mice and VD3 deficient mice, though coupling VD3 deficiency with Af-CRS did not exacerbate CD4 or T-regulatory cells numbers. Lastly, VD3 deficiency had a modifying or exacerbating impact on nasal lavage levels of IFN-γ, IL-6, IL-10 and TNF-α, but had no impact on IL-17A.

**Conclusions:**

VD3 deficiency causes changes in sinonasal immunity, which in many ways mirrors the changes observed in Af-CRS mice, while selectively exacerbating inflammation. Furthermore, both VD3 deficiency and Af-CRS were associated with altered sinonasal VD3 metabolism causing reductions in local levels of the active VD3 metabolite, 1,25(OH)_2_D_3_, even with adequate circulating levels.

## Introduction

Chronic rhinosinusitis (CRS) affects up to 16% of the United States population and has few proven treatments [1]. CRS represents one disease that is composed of a wide variety of clinical phenotypes. CRS with nasal polyps (CRSwNP) is the most difficult form of the disease to treat with greater than 50% of patients going on to have multiple surgeries in their lifetime [2]. Further complicating treatments for CRSwNP is that 48% of these patients also have a concurrent diagnosis of asthma [3] and 80% are atopic [1]. CRSwNP is characterized by a polarized type 2 microenvironment [4], which drives many of the physical symptoms associated with this disease such as rhinorrhea, mucus production and tissue remodeling [5, 6].

Vitamin D3 (VD3), also known as cholecalciferol, is a naturally occurring secosteroid hormone, which shares a number of anti-inflammatory functions with corticosteroids [7] and can regulate the functions of a broad range of immune and non-immune cell types [8, 9]. VD3 synthesis begins in the skin where pro-VD3 is metabolized to pre-vitamin D3. Following binding to vitamin D binding protein, it is transported to the liver and metabolized to 25-hydroxyvitamin D3 [25(OH)D_3_]. VD3 is also the form of vitamin D found in many over the counter supplements and used in the fortification of food products such as cereal and milk. In the final step of its metabolism, 1α-hydroxylase converts 25(OH)D_3_ to its metabolically active form, 1α,25-dihydroxyvitamin D_3_ [1,25(OH)_2_D_3_]. 1,25(OH)_2_D_3_ then binds to the VD3 receptor (VDR) which is expressed on nearly all cells in the body [10]. Originally it was thought that conversion of 25(OH)D_3_ to the active metabolite 1,25(OH)_2_D_3_ occurred exclusively in the kidneys, but studies have recently shown that 1,25(OH)_2_D_3_ can be generated locally by a variety of cell types in the upper and lower airways [11–14].

We have reported, and subsequent studies by others have confirmed, that the majority of patients with CRSwNP are 25(OH)D_3_ deficient (<32 ng/ml) as compared to controls or CRS without nasal polyp patients [14–23]. These 25(OH)D_3_ deficiencies in patients with CRSwNP were independent of age, race, gender, atopic status, body mass index and diagnosis of asthma [14–16, 24]. Furthermore, systemic 25(OH)D_3_ levels inversely correlated with disease severity as measured by Lund-Mackay CT scoring [22]. Systematic reviews have also found a significant relationship between low 25(OH)D_3_ levels and increased inflammation in patients with CRSwNP [18, 25]. Similarly, studies in the lower airway have also demonstrated a potential role of VD3 in the regulation of chronic airway inflammation. In a study of children with asthma, higher 25(OH)D_3_ levels were associated with reduced likelihood for hospitalization for asthma-related complications and reduced use of anti-inflammatory medications [26]. In steroid-resistant asthmatics, it has been shown that VD3 administration can reduce Th2 skewing [27]. While numerous human studies have described an association between VD3 deficiency and changes in disease severity and inflammation, the impact of deficiency on regulation of inflammation in the upper airway has not been explored.

In these studies, we address this question by examining the impact of dietary VD3 deficiency utilizing a previously characterized murine model of CRS that employs intranasal delivery of *Aspergillus fumigatus* (Af) to induce sinonasal airway inflammation, similar to what is observed in patients with CRSwNP or allergic fungal rhinosinusitis (AFRS), a subset of CRSwNP with known fungal allergies [28–31]. Af is a ubiquitous fungus that contains multiple antigens and is the most common fungus in the airway [32, 33]. Furthermore, fungal allergies and/or hyper-responsiveness to fungal antigens are hypothesized to contribute to pathogenesis of CRS [34]. Using this murine model of *Af-*induced CRS (Af-CRS), we examined the impact of dietary VD3 deficiency on sinonasal immune cell infiltrate, type 1 and type 2 cytokine levels and respiratory outcomes. Sinonasal VD3 metabolism and signaling were also examined given prior reports that patients with CRSwNP have an impaired sinonasal ability to metabolize 25(OH)D_3_ to 1,25(OH)_2_D_3_ due to a downregulation in epithelial cell expression of CYP27B1, and its respective enzyme,1α-hydroxylase [35–37]. In these studies, we will describe that while VD3 deficiency can, in many cases, exacerbate Af-CRS, VD3 deficiency alone causes profound changes in sinonasal immunity. We also demonstrate the novel finding that the chronic inflammation associated with Af-CRS is capable of reducing sinonasal 1,25(OH)_2_D_3_ levels even in presence of adequate circulating stores of 25(OH)D_3_ and 1,25(OH)_2_D_3_.

## Methods

### Murine model of atopic CRS

All studies were approved by the Medical University Institutional Animal Care and Use Committee and conducted following the Animal Research: Reporting In Vivo Experiments (ARRIVE) Guidelines (see **S1 File–NC3Rs ARRIVE Guidelines Checklist**). Eight week old female Balb/c mice were purchased from Jackson Laboratories. Study mice were given food lacking VD3 (Harlan Tekland, Madison, WI, TD 89123) or control diet (VD3 replete) (TD 89124). Diets were matched for all nutrients, except VD3 (see **Table A in S2 File**). Mice were fed control of VD3 deficient diet for 4 weeks to establish 25(OH)D_3_ deficiency, which was verified by measuring serum 25(OH)VD3 levels with ELISA (ImmunoDiagnostic Systems, Fountain Hills, AZ).

For these studies we used a previously described murine model of Af induced-CRS [31, 38–41]. This model was selected as it mimics many of the hallmarks of human CRSwNP including sinonasal eosinophilia, type 2 inflammation, goblet cell hyperplasia, increased mucin production and loss of ciliated epithelium. Briefly, mice were sensitized via intraperitoneal injection with Af (50:50 mixture of mycelial extract and culture filtrate extract)(Greer Laboratories, Lenoir, NC), 200 μg absorbed in 2 mg of alum in 0.5 mL of phosphate buffered saline solution (PBS). Prior to use, Af was determined to be endotoxin free (<0.1 endotoxin units/mL) using a Limulus amebocyte lysate (LAL) endotoxin test (Thermofisher) [42]. Control mice received 2 mg of alum in 0.5 mL of PBS and were not sensitized to Af. One week after sensitization, mice were given an intranasal Af antigen challenge of 10 μg (5 μL/nares) of Af extract or 5 μL of PBS three times per week for four consecutive weeks. All mice were age matched at time of end point analysis and tissue collection.

### Mouse respiratory outcome measures

Dual-chamber, whole-body restrained plethysmography, utilizing mouse-sized plethysmography chambers, was used to assess upper airway functions (emka Technologies, France). The differential pressure was acquired by an acquisition amplifier at 1 kHz (usbAMP, emka Technologies) and processed in analysis software (IOX Base 2c, IOX 1 PULMO 2c, emka Technologies) [43]. Enhanced pause (Penh) was selected since changes in nasal cavity resistance can impact as much as 50% of total lung resistance as reflected by Penh [44]. Penh was calculated as the peak expiratory flow/peak inspiratory flow x Pause. Specific airway resistance (Sraw) was examined as it as it correlates with circulating IgE, a marker of type 2 inflammation [45] and is an indicator of nasal blockage in murine and guinea pig models [46–49].

### Nasal lavage collection and related measures

Nasal airway lavage fluid (NALF) was collected as previously described [50–53]. Briefly, partial tracheal resection was performed and a plastic catheter was inserted into the tracheal opening toward the upper airway. One milliliter of PBS was flushed through the upper airway, and collected in a tube situated outside the nostrils, and then preserved in ice prior to sample preparation. Cytospin and differential cells counts were conducted on NALF as our group has previously described [54]. Total protein was measured by bicinchoninic acid assay (BCA) according to the manufactures’ instructions (ThermoFisher Scientific, Waltham, MA) and was used as a readout of airway damage. NALF cytokine levels were measured using BD Biosciences Murine Th1/Th2/Th17 CBA kit. The limitation for detection for each of the cytokines (in pg/ml) is: IL-2 = 0.1; IL-4 = 0.03, IL-6 = 1.4, IFN-g = 0.5, TNF = 0.9, IL-17A = 0.08, IL-10 = 16.8.

### Blood collection and serum measurements

Blood was collected by cardiac puncture and serum collected to measure total IgE (BD Biosciences), 25(OH)D_3_ or 1,25(OH)_2_D_3_ (Imunnodiagnostic Systems, Gaithersburg, MD) by ELISA.

### Sinonasal tissue collection and processing for flow cytometric analysis

Sinonasal tissue was collected using a scalpel to make a sagittal incision through the skin and subcutaneous tissues extending from the tip of the nose to the vertex of the scalp. Blunt dissection was used to free the soft tissue envelope from the underlying skull and nasal bones. With the nasal bones fully exposed, a scalpel was used to incise along the frontonasal suture and sagittal suture to expose the nasal cavity. A cerumen hook and fine forceps were used to remove the sinonasal mucosa. Images showing the murine sites from which tissue was collected are shown in **Figure A in S2 File**. A single cell suspension was then obtained by mechanical separation, passed over a 70 μM cell strainer, and then rinsed twice with PBS.

Immediately following collection, sinonasal mucosa was immunostained and analyzed by flow cytometry. VDR and 1α-hydroxylase (ThermoFisher), which is encoded by CYP27B1, expression was examined by flow cytometry as we have previously described [35, 36, 55]. Flow cytometry was used so as to determine the cell specific expression of 1α-hydroxylase. Similar to our previous studies, FoxP3/transcription factor staining buffer (eBioscience/ThermoFisher) was used for VDR and 1α-hydroxylase staining, which allows for examination of cytoplasmic and nuclear expression of VDR and 1α-hydroxylase. 25-hydroxylase (CYP2R1) which converts VD3 to 25(OH)D3 was not examined due to prior reports in human sinonasal tissue that this gene was found to be expressed at near undetectable levels, and was not affected by sinonasal inflammatory disease [36]. Sinonasal epithelial cells were identified by positive staining for EpCam. To determine the presence of specific subsets of immune cells, we used methods similar to those we have previously reported in human sinonasal tissues [15, 35, 56–59]. DC subsets were determined by their expression of the following markers: moDCs (CD11b+, CD206+, CD209+), pDC (PDCA-1+, Siglec-H+, CD11b^-^); CD11b- cDC (CD11b-, CD11c+, CD103+) and CD11b+ cDC(CD11b+, CD11c+, CD115+). T-cells were identified by their staining for CD4, CD8, or in the case of T-regulatory cells (T-regs) staining for CD4, CD25 and FoxP3. Matched isotype controls were used for each stain. 7AAD positive cells (dead cells) were excluded from analysis. Representative gating plots and gating strategies are shown for DCs in **Figure** C **in S2 File** and T-cells in **Figure D in S2 File.** Cytometric analysis was performed using a Guava 8HT flow cytometer and data analyzed using FCS Express 5 (De Novo Software, Glendale, CA).

### Sinonasal analysis of 25(OH)D_3_ and 1,25(OH)_2_D_3_ levels

To account for the short half-life of 1,25(OH)_2_D_3_ (2–3 hours), immediately following tissue collection, sinonasal mucosa lysates were prepared and assessed for 25(OH)D_3_ and 1,25(OH)_2_D_3_ by ELISA as previously described [35, 36]. To correct for potential variations that could be caused by the amount of tissue and/or protein present, all final sinonasal 25(OH)D_3_ and 1,25(OH)_2_D_3_ values were corrected for by total protein, as measured by the BCA method.

### Statistics

Statistical analysis was conducted using GraphPad Prism 6.0 software (La Jolla, CA). A D'Agostino & Pearson omnibus test was used to determine if data sets were normally distributed, all of which were found to be. For data sets containing only two groups an unpaired student t-test was used. For data in which there were four groups, a one-way ANOVA with post-hoc Holm-Sidak's multiple comparisons test was used to determine statistical significance between specified data pairs. *p* values <0.05 were considered significant. Values shown are mean ± standard deviation.

## Results

### Dietary VD3 deficiency and Af-CRS are associated with worsened respiratory outcomes and airway damage

**Fig 1:**
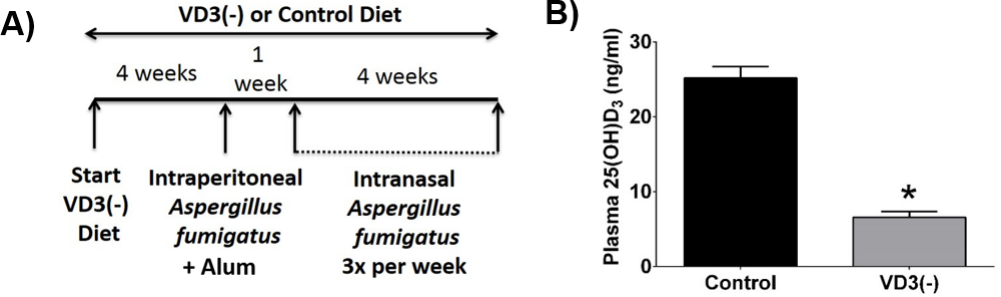
Model summary and plasma 25(OH)D_3_ levels. (**A)** Schematic of the vitamin D deficient Af-CRS protocol. **B)** Confirmation of 25(OH)D_3_ deficiency after 4 weeks on VD3 deficient diet. n = 4 control, 6 vitamin D3 deficient [VD3(-)] mice per group. * p<0.05 vs control.

In these studies, we used a previously characterized murine model of atopic CRS that utilizes intranasal delivery of Af to induce upper respiratory tract inflammation that is similar to humans with CRSwNP or AFRS [38–40]. Prior to initiation of the model, mice were fed VD3 deficient or control diet for 4 weeks. A summary of the model timeline is shown in **Fig 1A**. Systemic 25(OH)D_3_ deficiency was confirmed after 4 weeks, at which time levels of 25.2±1.8 and 6.7±0.8 ng/ml were observed in VD3 replete vs deficient mice, respectively (**Fig 1B**).

**Fig 2:**
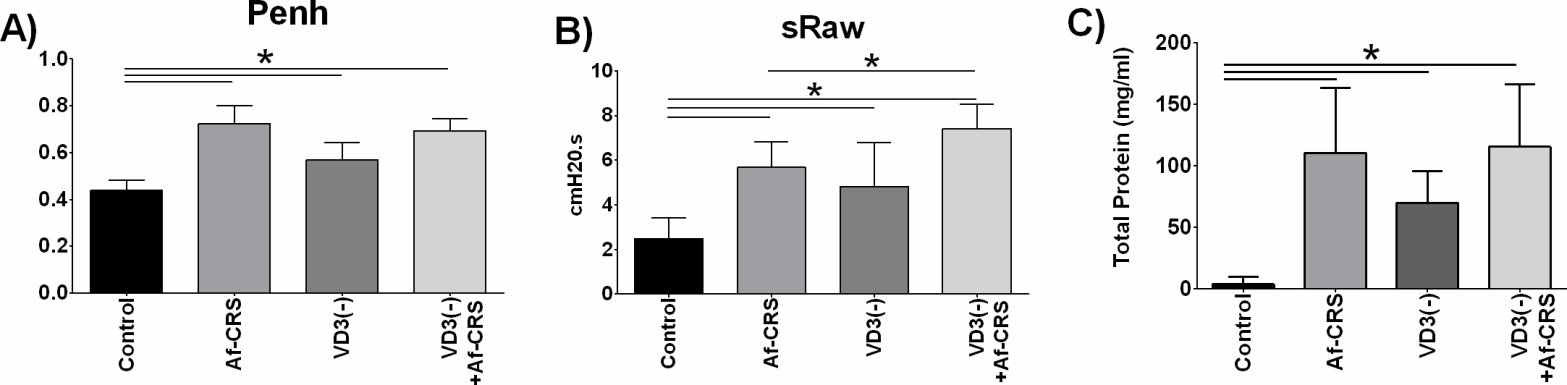
VD3 deficiency alone worsens airway function and injury similar to Af-CRS. **(A)** Penh and **(B)** sRaw were measured by dual-chamber whole-body restrained plethysmography 24 hours after the last Af i.n. instillation. (**C**) Nasal lavage total protein was measured as an indicator of upper airway damage. *p<0.01 vs control. # p<0.05 vs Af-CRS. n = 5–6 mice/group.

Given the strengths and limitations of the various respiratory outcome measures in mice [60], we elected to use both Penh and sRaw as measured by restrained dual-chamber restrained whole-body plethysmography 24 hours after the final Af treatment. As shown in **Fig 2A and 2B**, mice with Af-CRS alone had increased Penh and sRaw, respectively. Similar to Af-CRS mice, those with VD3 deficiency alone also had increased Penh and sRaw. Comparisons between mice with Af-CRS or VD3 deficiency, found no significant difference in either Penh or sRaw. Lastly, the combined effects of VD3 deficiency plus Af-CRS resulted in increased Penh and sRaw as compared to controls, though only sRaw was found to be exacerbated in comparison to Af-CRS alone. In mice with VD3 deficiency alone, as compared to VD3 deficiency plus Af-CRS, no difference in Penh was observed. However, sRaw was significantly elevated in mice with both VD3 deficiency and Af-CRS, as compared to VD3 deficiency or Af-CRS alone.

Given that these airway function results suggested nasal pathology, we next measured NALF total protein levels as a means to assess airway injury. Compared to controls, mice with Af-CRS alone or VD3 deficiency alone had increased total protein levels in their NALF (**Fig 2C**). VD3 deficiency coupled with Af-CRS was similar to either condition alone. Together, these results suggest that VD3 deficiency alone can adversely impact upper airway function and promote airway injury that is not dissimilar to having Af-induced CRS.

### Sinonasal 25(OH)D_3_ metabolism, but not VDR expression, is altered by VD3 deficiency or Af-CRS

**Fig 3:**
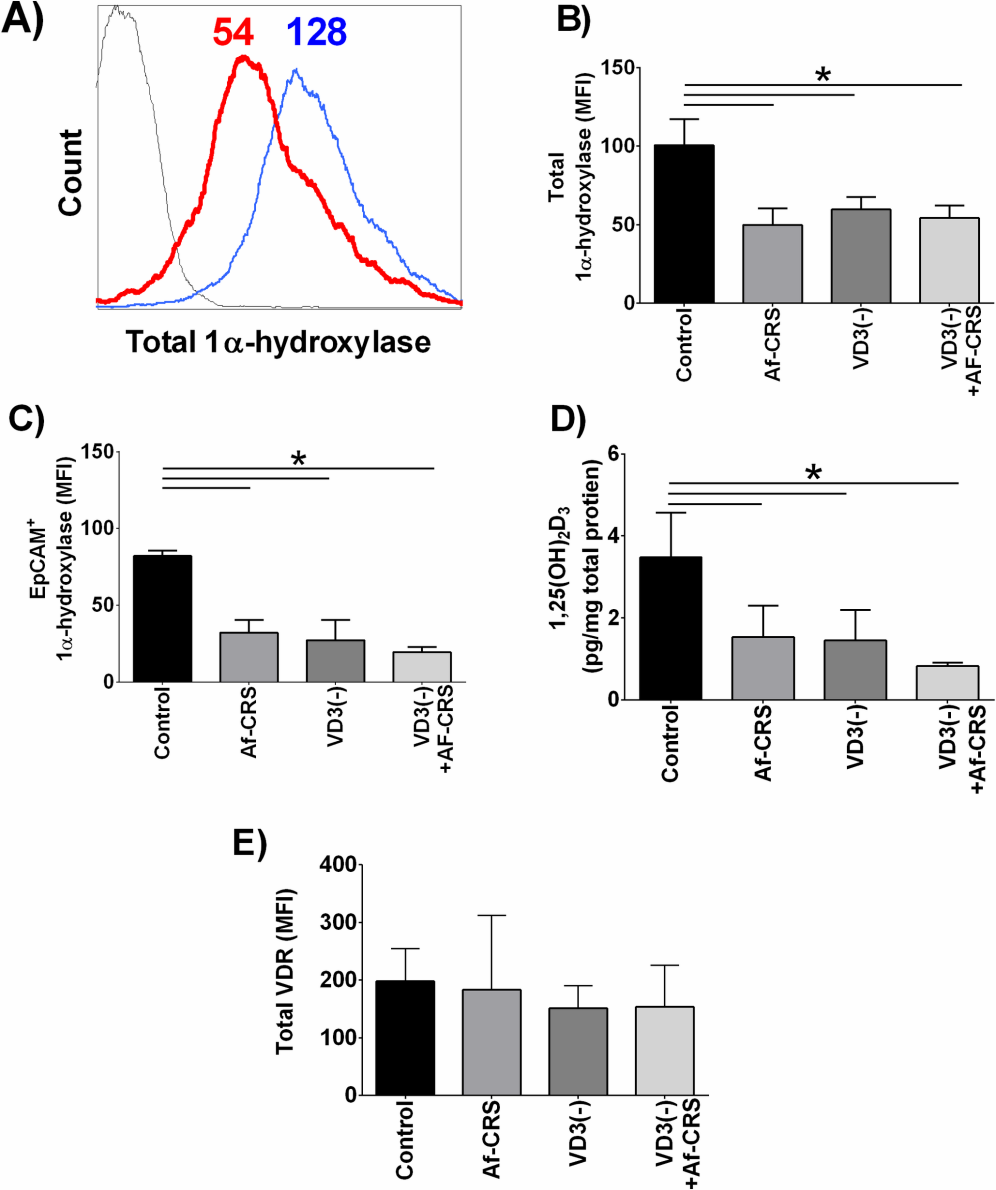
VD3 deficiency and Af-CRS are both associated with reduced sinonasal tissue levels of 1α-hydroxylase and 1,25(OH)_2_D_3_. (**A**) Overlays of representative histograms of total sinonasal 1α-hydroxylase staining. Histogram colors represent: black = isotype control, red = Af-CRS 1α-hydroxylase staining, blue = control 1α-hydroxylase. Values shown are the mean fluorescent intensity (MFI) for each of the peaks shown. Quantification of (**B**) total 1α-hydroxylase and (**C**) epithelial cell specific 1α-hydroxylase expression were measured by flow cytometry and are reduced in all groups compared to controls. (**D**) Sinonasal tissue lysates examined for 1,25(OH)_2_D_3_ by ELISA and corrected for total protein. Compared to controls, 1,25(OH)_2_D_3_ was reduced in all groups. (**E**) Total sinonasal VDR expression was measured by flow cytometry and was found to be the same amongst all groups. n = 4 mice/group. *p<0.05 between indicated groups.

Patients with CRSwNP and AFRS have been shown to have reduced circulating and sinonasal levels of 25(OH)D_3_. Furthermore, they display reduced sinonasal levels of 1α-hydroxylase and consequently low 1,25(OH)_2_D_3_ [35, 36]. Due to these clinical studies we sought to examine if local sinonasal VD3 metabolism was affected in our rodent model of disease. As shown by representative histogram in **Fig 3A** and quantified in **Fig 3B,** compared to controls, Af-CRS mice had reduced total 1α-hydroxylase. Similar to humans [35, 36], sinonasal epithelial cells were the predominate source accounting for an average of 82% of 1α-hydroxylase^+^ cells (**Fig 3B and 3C**). Furthermore, compared to control mice sinonasal epithelial cell-specific 1α-hydroxylase was reduced in mice with Af-CRS (**Fig 3C**). VD3 deficiency alone also caused a reduction in sinonasal epithelial expression of 1α-hydroxylase. VD3 efficiency coupled with Af-CRS did not alter epithelial cells 1α-hydroxylase levels compared to either group alone. Consistent with the reduction in sinonasal 1α-hydroxylase, we also observed that Af-CRS mice had reduced sinonasal levels of 1,25(OH)_2_D_3_ as compared to control mice (**Fig 3D**). Nearly identical to mice with Af-CRS, VD3 deficient mice also had reduced sinonasal 1α-hydroxylase and 1,25(OH)_2_D_3_ levels. The combined effect of VD3 deficiency with Af-CRS did not exacerbate changes in local VD3 metabolism when compared to Af-CRS or VD3 deficiency alone. Lastly, total sinonasal VDR expression was examined by flow cytometry in which no difference between any of the groups was detectable (**Fig 3E**).

**Fig 4:**
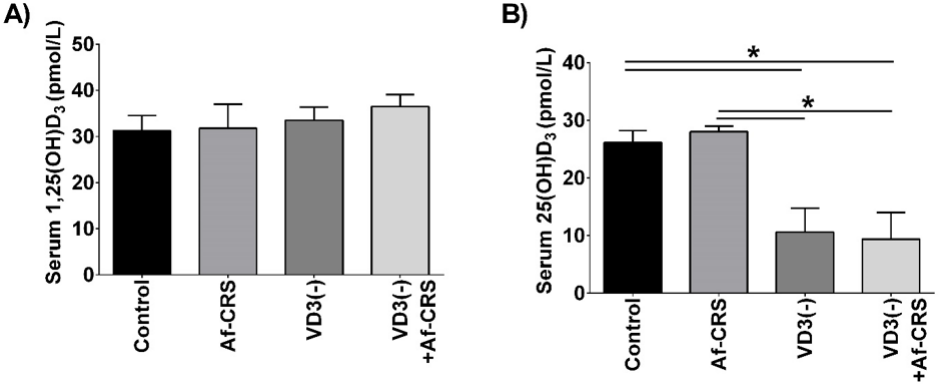
Af-CRS does not alter serum levels of 1,25(OH)_2_D_3_ or 25(OH)D_3_. **(A)** Serum 1,25(OH)_2_D_3_ levels were not significantly different amongst groups, regardless of disease or dietary VD3 status. (**B)** Serum 25(OH)D_3_ levels are not impacted by local inflammation associated with Af-CRS. As expected, mice with dietary VD3 deficiency demonstrate reduced circulating 25(OH)D_3_ levels. N = 4 mice/group. *p<0.05 between indicated groups.

**Fig 5:**
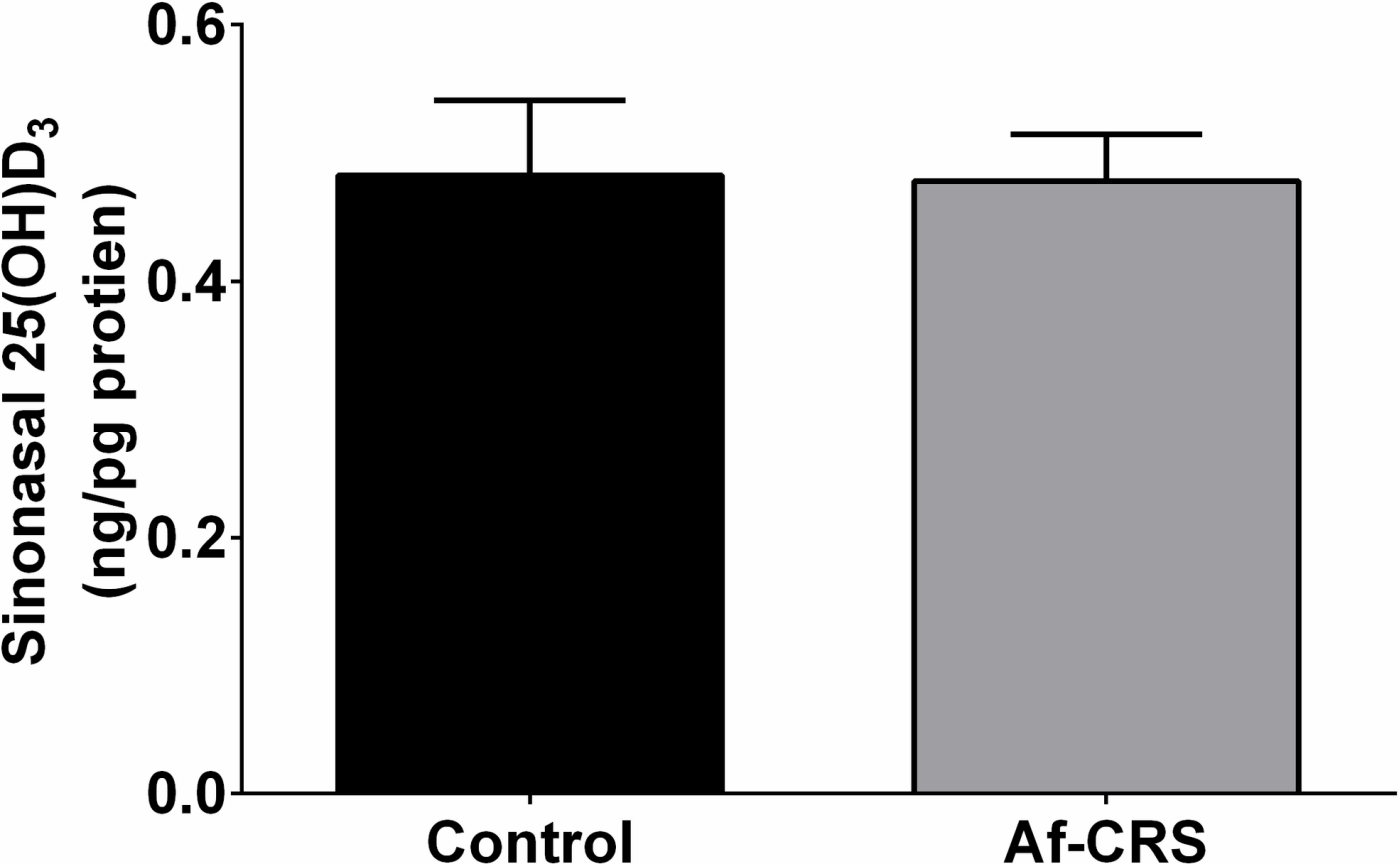
Af-CRS is not associated with sinonasal reductions in 25(OH)D_3_. Sinonasal levels of 25(OH)D_3_ were measured by ELISA and results normalized to total protein concentration. Both groups were fed control diet and were systemically 25(OH)D3 sufficient. N = 3 mice/group.

To confirm that the changes in local 1,25(OH)_2_D_3_ were not the result of a reduced circulating supply, we also examined plasma 1,25(OH)_2_D_3_ levels. Comparable to humans with or without CRS [35], no differences in circulating levels of 1,25(OH)_2_D_3_ levels was observed between any of the groups (**Fig 4A**). Circulating 25(OH)D3 levels were not reduced in mice with Af-CRS, but were reduced in those with dietary deficiency compared to controls (**Fig 4B**). Furthermore, there were no differences in circulating or sinonasal levels of 25(OH)D_3_ between control mice and those with Af-CRS (**Fig 5**). Collectively, these novel findings demonstrate that VD3 deficiency and the inflammation associated with Af-CRS are capable of reducing sinonasal levels of the active metabolite of VD3, 1,25(OH)_2_D_3_, without any change to circulating 25(OH)D_3_ or 1,25(OH)_2_D_3_ levels.

### VD3 deficiency, both alone and in combination with Af-CRS, selectively modifies the sinonasal immune cell profile

**Fig 6:**
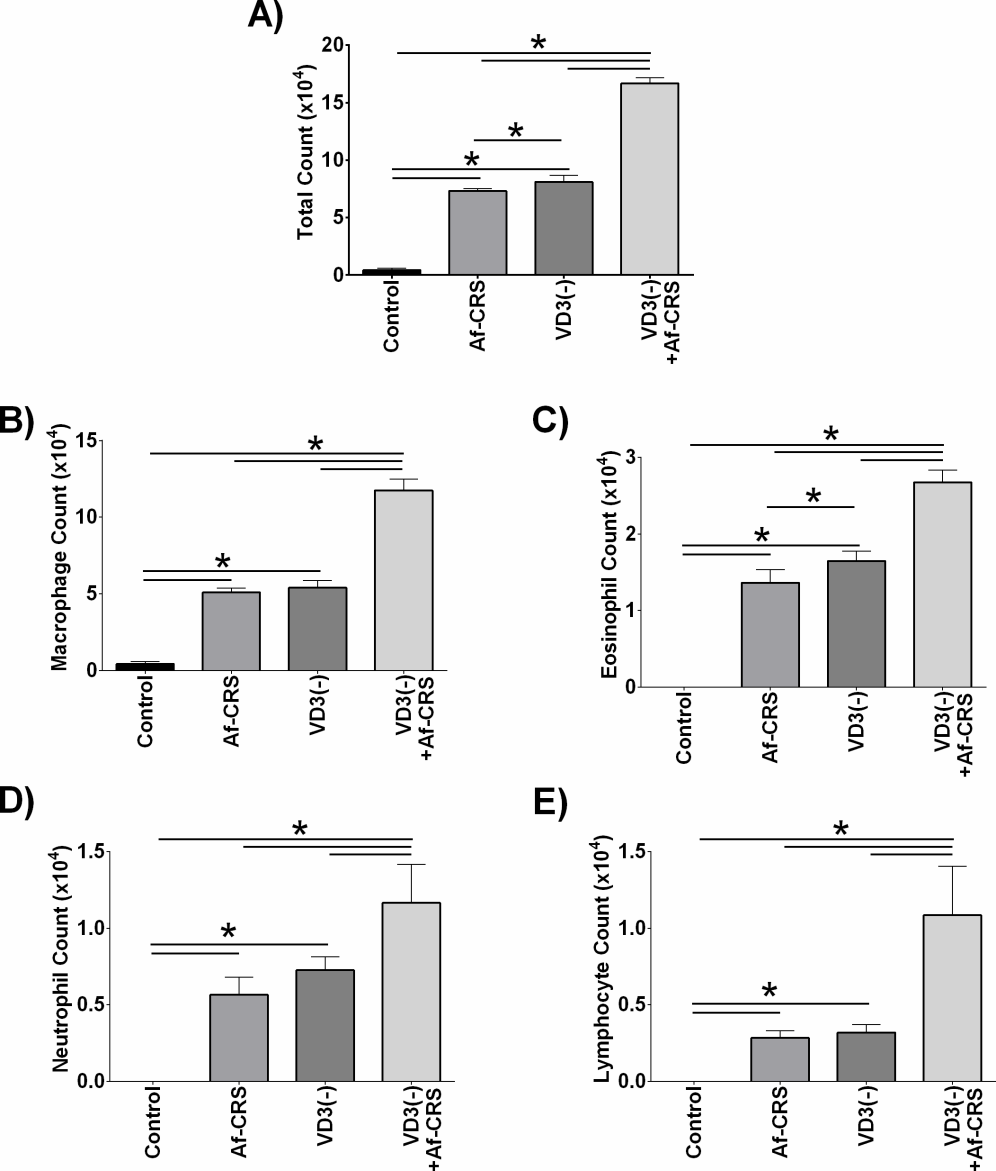
VD3 deficiency exacerbates changes in sinonasal immune cell infiltrate. Both Af-CRS and VD3 deficiency were associated with increased lavage cell counts of (**A**) total cells, (**B**) macrophages, (**C**) eosinophils, (**D**) neutrophils, and (**E**) lymphocytes. *p<0.05 between indicated groups. n = 4 mice/group.

Next, we examined the impact of VD3 deficiency on local sinonasal inflammation by assessing the presence of immune cells in NALF. When compared to controls, total cell count (**Fig 6A**) as well as macrophages (**Fig 6B**), eosinophils (**Fig 6C**), neutrophils (**Fig 6D**) and lymphocytes (**Fig 6E**) were all increased in mice with Af-CRS alone. Neutrophils and lymphocyte numbers were similar between mice with VD3 deficiency alone and Af-CRS alone. However, eosinophils were the only cell type that demonstrated a modest, but statistically significant, increase in cell numbers in VD3 deficient mice as compared to those with Af-CRS. VD3 deficiency plus Af-CRS was found to exacerbate increases in cell numbers as compared to Af-CRS alone or VD3 deficiency alone for each of the cell types examined by differential count. Collectively, these data demonstrate that VD3 deficiency exacerbates inflammation associated with Af-CRS.

**Fig 7:**
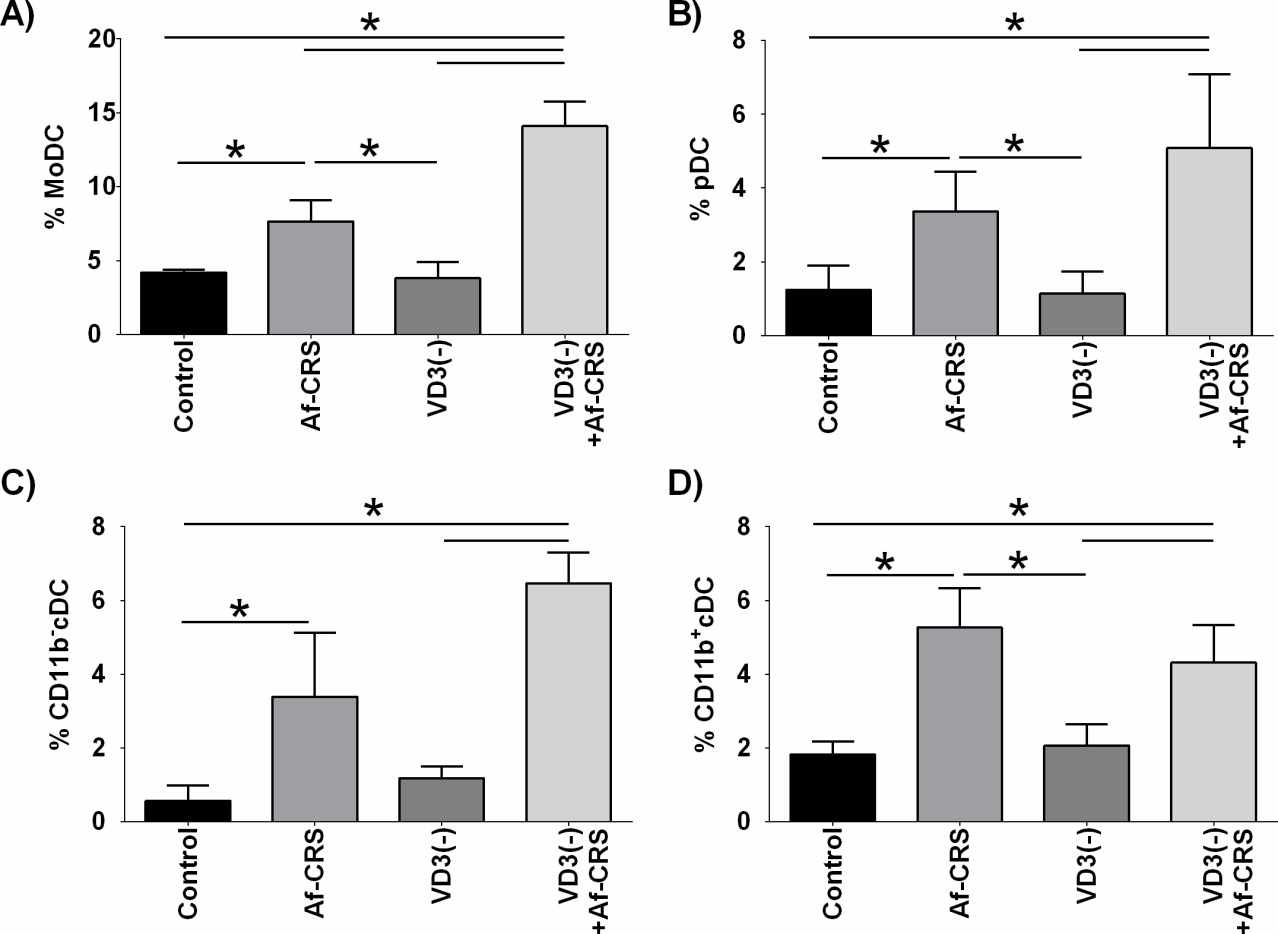
VD3 deficiency exacerbates increases in sinonasal moDCs, associated with Af-CRS. DC subsets were defined by the following marker combinations; moDCs = CD11b+, CD206+, CD209+, pDC = CD11b-, PDCA-1+, Siglec-H+, cDC (CD11b-) = CD11b-, CD11c+, CD103+, cDC (CD11b+) = CD11b+, CD11c+, CD115. Dead cells excluded by 7AAD. n = 4/per group. *p<0.05 between indicated groups.

Given the robust impact of VD3 deficiency on the nasal lavage immune cell numbers, we next focused on identifying specific cell types found in the sinonasal mucosa that could not be determined solely by differential counts. Given previous reports showing that VD3 deficiency is associated with changes in DCs in patients with CRS [14, 16], we first examined the presence of various sinonasal DC subsets. As shown in **Fig 7A–7D**, as compared to controls, mice with Af-CRS alone displayed increases in local sinonasal levels of each of the DC subsets analyzed. Conversely, VD3 deficiency alone did not alter the percentage of any of the studied sinonasal DC subsets, as compared to controls. Lastly, moDCs were the only DC subset in which VD3 deficiency plus Af-CRS led to an increase in local sinonasal infiltrates as compared to mice with Af-CRS alone (**Fig 7A**).

**Fig 8:**
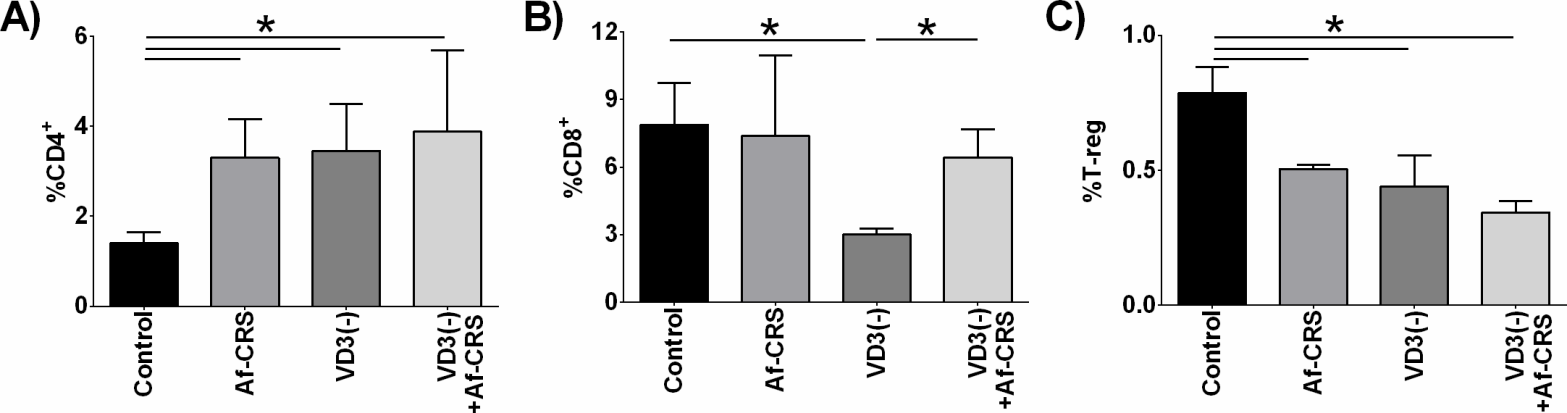
VD3 deficiency alone causes changes in sinonasal CD4 and T-regs similar to Af-CRS. Sinonasal (**A**) CD4, (**B**) CD8 and (**C**) T-regulatory cells (CD4^+^CD25^+^FoxP3^+^) were identified by immunostaining and flow cytometric analysis. *p<0.05 between indicated groups. n = 4-5/group.

Next we examined the impact of VD3 deficiency on sinonasal T-cell subsets. As shown in **Fig 8A**, when compared to sinonasal tissue from control mice, CD4+ T-cells were found to increase in all three experimental groups. Comparison between mice with Af-CRS versus VD3 deficiency found similar numbers of CD4+ T-cells. VD3 deficiency coupled with Af-CRS did not alter sinonasal percentages of CD4+ T-cells as compared to Af-CRS alone. With regard to CD8+ T-cells, only mice with VD3 deficiency were found to have a reduced percent of sinonasal CD8+ T-cells (**Fig 8B**). Yet, mice with VD3 deficiency coupled with Af-CRS had CD8+ T-cell numbers similar to controls. Lastly, we examined the presence of sinonasal T-regs. Compared to controls, Af-CRS alone or VD3 deficiency alone had significant reductions in T-regs cells as compared to controls (**Fig 8C**). VD3 deficiency coupled with Af-CRS was also associated with reduced sinonasal T-regs as compared to control, however there was no difference as compared to Af-CRS or VD3 deficiency alone. Together, these results demonstrate that VD3 deficiency has a profound impact on sinonasal immune composition which in many ways is similar to changes seen in mice with Af-CRS.

### VD3 deficiency modifies changes in sinonasal pro-inflammatory, Type 1 and Type 2 cytokines in mice with Af-CRS

**Fig 9:**
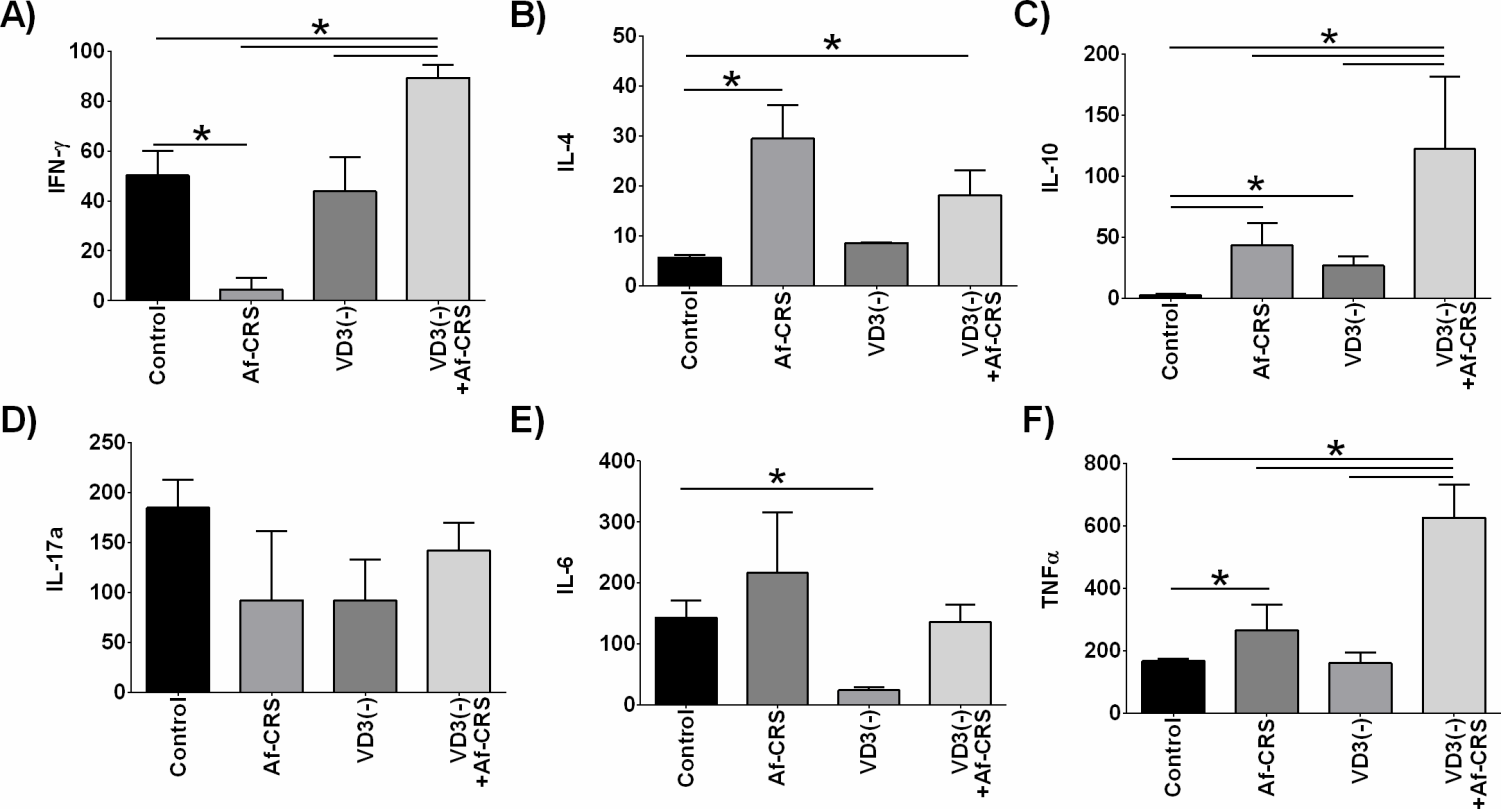
VD3 deficiency selectively exacerbates nasal lavage type 1, type 2 and pro-inflammatory cytokines. NALF cytokines were measured by cytometric bead array. All values shown are in pg/ml. *p<0.05 between indicated groups. n = 3–4 mice per group.

Lastly, we examined the local impact of VD3 deficiency on inflammatory cytokine levels in NALF. While Af-CRS was associated with reduced levels of the type 1 cytokine, IFN-γ, on the other hand Af-CRS with VD3 deficiency caused significant elevation in IFN-γ as compared to all other groups (**Fig 9A**). VD3 deficiency alone had no impact on IFN-γ as compared to control. Examination of the type 2 cytokines, IL-4 and IL-10, showed that Af-CRS was associated with increased sinonasal lavage IL-4, whereas no change in mice with VD3 deficiency was noted as compared to controls (**Fig 9B**). The combination of VD3 deficiency with Af-CRS, while associated with increased IL-4 versus controls, was no different than mice with Af-CRS alone. Compared to controls, mice with Af-CRS or VD3 deficiency had increased IL-10 (**Fig 9C**). The combined effect of VD3 deficiency and Af-CRS was associated with a synergistic increase in IL-10 that was significantly higher than all other groups. There were no statistically significant differences between any of the groups for sinonasal IL-17a levels (**Fig 9D**). The only statistically significant change in IL-6, was a reduction in levels in mice with VD3 deficiency compared to controls (**Fig 9E**). Lastly, the pro-inflammatory cytokine TNF-α was examined (**Fig 9F**). Compared to control mice, those with Af-CRS had increase NALF levels of TNF-α, while VD3 deficiency had no effect. VD3 deficiency along with Af-CRS resulted in significantly elevated TNF-α levels, compared to all groups.

As an additional local marker of Th2 inflammation, we measured circulating total IgE. Not surprisingly, we observed that mice with Af-CRS had increased total IgE as compared to control mice (**Figure D in S2 File**). VD3 deficiency had no impact on circulating IgE, either alone or in combination with Af-CRS. Taken together, these results suggest that VD3 deficiency can have both a modifying and exacerbating role in the production of type 1, type 2, and pro-inflammatory cytokines, though it does not effect on circulating IgE levels.

## Discussion

In the current studies, we sought to identify how VD3 deficiency impacts local sinonasal immunity in Af-CRS, as a means to provide potential mechanistic insights to prior observational human studies showing that VD3 deficiency is associated with more severe CRSwNP [22]. While we hypothesized that VD3 deficiency would exacerbate immunological changes associated with Af-CRS, this was found to hold true to a limited number of factors. Unexpectedly, we clearly demonstrate that VD3 deficiency alone has a profound impact on sinonasal immunity and in many cases displays an immune phenotype that was not dissimilar to mice with Af-CRS.

One important area that has not been previously described in murine upper or lower airway models in vivo is the impact of allergic inflammation on local VD3 metabolism. Studies in tuberculosis, cancer and sarcoidosis have established a critical role for local 25(OH)D_3_ metabolism in disease progression [61–66]: a role we do not understand in CRSwNP or asthma. Here we demonstrate that mice with Af-CRS or VD3 deficiency alone had reduced sinonasal levels of 1α-hydroxylase and 1,25(OH)_2_D_3_. This reduction is similar to the impaired sinonasal metabolism observed in patients with CRSwNP or AFRS [35, 36].

Similar to humans, in our rodent studies we also demonstrated that sinonasal epithelial cells were the major source of sinonasal 1α-hydroxylase [15]. With regard to 1α-hydroxylase expression, our results differ from those using the transformed, lung 16HBE cell line, which described 1α-hydroxylase as being increased in response to *A*. *fumigatus* conidia [67]. These differences could be caused by the additional presence of mycelia in our studies or differing responses by the airway cell type being studied. Another important difference between the upper and lower airway that could be responsible for these varying results is that while lung epithelial cells can metabolism VD3 to 25(OH)D_3_ [68], upper airway epithelial cell do not express CYP2R1, and as such, cannot metabolize VD3 [36].

While the cause of 25(OH)D_3_ deficiency in CRSwNP patients is unknown, Af-CRS mice, similar to mice with house dust mite-induced airway inflammation, had no reduction in systemic 25(OH)D_3_ [69], suggesting that local inflammation does not alter systemic VD3 to 25(OH)D3 metabolism. Also similar to humans, we found that circulating deficiencies in 25(OH)D_3_ were associated with sinonasal deficiencies as well [36]. We did observe that Af-CRS mice had sinonasal, but not systemic 1,25(OH)_2_D_3_, deficiencies compared to controls supporting prior human studies that circulating 1,25(OH)_2_D_3_ was not reflective of sinonasal tissue levels [35]. These results are similar to previous reports in placenta tissue showing that 25(OH)D_3_ deficiency causes a down regulation in *CYP27B1*, the gene that encodes for 1α-hydroxylase [70]. Taken together our results demonstrate that **1)** the chronic inflammation associated with CRS is capable of impairing local 25(OH)D3 metabolism and that **2)** adequate circulating levels of 1,25(OH)_2_D_3_ are not sufficient to overcome reductions in sinonasal metabolism.

The discrepancy between VD3 deficiency being associated with more severe disease and the apparent failure of systemic supplementation remains the subject of much debate [20, 71–73]. While a number of reports have linked VD3 deficiency to worsened clinical outcomes, the use of oral VD3 supplementation has yielded mixed results for the treatment of airway diseases, primarily asthma [74–79]. Most recently the results of the VIDA trial (VD3 Add-on Therapy Enhances Corticosteroid Responsiveness in Asthma) demonstrated that VD3 supplementation did not improve patient outcomes, including in patients who also had sinusitis [80, 81]. Recently it has been shown the VD3 supplementation failed to improve clinical outcomes or antifungal efficacy for patients with invasive aspergillosis [82]. As we have demonstrated in these studies one possible explanation for the failure of oral VD3 treatment to improve clinical outcomes may be caused by an impaired local metabolism of 25(OH)D_3_ to 1,25(OH)_2_D_3_ in the airway. Thus, it is likely that to most effectively utilize VD3 supplementation, we must have adequate circulating 25(OH)D_3_ as well as appropriate levels of 1α-hydroxylase to allow for the local metabolism of 25(OH)D_3_ to 1,25(OH)_2_D_3_. Murine studies that have attempted to address the ability of VD3 supplementation after deficiency have yielded mixed results. While supplementation reduced eosinophil and neutrophil numbers in bronchoalverolar lavage [83], deficiency correction had only limited effect of improving epithelial integrity in the lung which was only modestly impacted by VD3 deficiency in otherwise healthy mice [84]. Collectively, these reports and our data highlight the need for additional research into how 25(OH)D3 deficiency and VD3 supplementation regulates local vitamin D metabolism and subsequent airway functions.

With regards to VDR expression, we did not see any change in sinonasal VDR expression with disease, VD3 deficiency or the combination of the two. These results are in agreement with prior reports that 25(OH)D_3_ status was not associated with any changes in sinonasal VDR expression gene expression in control subjects or those with CRSwNP [36, 37]. However, these results differ from those presented in the murine lung models which demonstrate a reduction in lung VDR expression in mice with a dietary VD3 deficiency [85]. Other in vitro reports that have shown that *A*. *fumigatus* down regulated cystic fibrosis-patient derived epithelial cell and macrophage VDR [86]. Because we looked at total and not cell specific differences in VDR, this may explain changes in VDR expression, it may be possible that VDR may be regulated differentially on different cells types, and would be an area of future investigation. However, at least in the upper airway, because there is no change in VDR expression with disease or VD3 deficiency it is possible that should local 1,25(OH)_2_D_3_ levels be restored to control levels, ample VDR expression would be present to promote activation of VD3-related signaling pathways in the upper airway.

To measure murine disease severity via respiratory function, we used Penh and sRraw as indicators of respiratory functions and nasal obstructions. In our model we saw sRaw, but not Penh, increase in mice with VD3 deficiency plus Af-CRS versus Af-CRS alone. One possible explanation for this discrepancy is that increased sRaw is also an indicator of nasal blockage unlike Penh [46–48]. Our results are consistent with studies examining lower airway function in which VD3 deficiency was found to increase airway responsiveness in mice with ovalbumin (OVA) induced inflammation as compared to mice that were VD3 replete [87]. However, it is important to point out that these studies do not directly equate to our use of Penh and sRaw and as they were done with methacholine challenge. Both studies do suggest that VD3 deficiency can adversely impact airway functions. With regards to the mechanism driving the impaired airway function, it is unlikely that one singular cell or process is responsible for the altered airway functions of mice with VD3 deficiency given that VDR is expressed on nearly every cell in the body. For example, VD3 has been shown regulate airway Th1/Th2 skewing, steroid responsiveness, lung growth and development, fibroblast and smooth muscle cell proliferation and epithelial-to-mesenchymal transition just to name a few of its effects [20].

Differential analysis of murine nasal lavage revealed that VD3 deficiency alone causes an increase in total nasal lavage inflammatory cell infiltrate, including elevations in macrophages, eosinophils, neutrophils and lymphocytes. Similar to previous reports in the lungs of OVA challenged mice [83, 87], we observed that VD3 deficient Af-CRS mice displayed increasing nasal lavage eosinophil numbers as compared to VD3 replete mice. Human studies in asthma have also demonstrated that VD3 deficiency is associated with increased sputum eosinophilia [88]. To our knowledge no reports have described the impact of VD3 deficiency on sinonasal eosinophil numbers in humans with sinusitis, though we have previously shown no association exists between 25(OH)D_3_ and eosinophilia systemically [22]. Moreover, we observed that VD3 deficiency exacerbated changes in neutrophil and lymphocyte numbers associated with Af-CRS. Our results also differed in that we observed increases in each of these cell populations with VD3 deficiency alone compared to control mice [83, 87]. VD3 deficiency also exacerbated increases in macrophage numbers in NALF. Macrophages have been shown in the lung to be an important local source of 1,25(OH)_2_D_3_, the synthesis of which is driven by TNF-α and IFN-γ [89, 90]. However, while we show an elevation of both of these cytokines in VD3 deficient mice with Af-CRS, at the same time we a saw decrease in sinonasal 1,25(OH)_2_D_3_ levels. This suggests that upper airway local production of 1,25(OH)_2_D_3_ may be more dependent on epithelial cells, which account for 80% of 1α-hydroxylase positive cells, and warrants future investigations.

One cell population that is susceptible to regulation by 25(OH)D_3_ is DCs [91], and as such were the focus of investigation in these studies. Similar to the increase in moDCs observed in patients with CRSwNP versus controls [14, 16, 58], we also observed that mice with Af-CRS have increased sinonasal moDCs. While VD3 deficiency alone had no impact on sinonasal moDCs, VD3 deficiency coupled with Af-CRS did lead to a significant increase in moDCs as compared to Af-CRS alone. These data lend some causation to previous observational studies which demonstrated an inverse correlation between 25(OH)D_3_ and moDCs in patients with CRSwNP [14, 16]. Given that moDCs have been shown to be associated with more severe Th2 inflammation and are elevated with 25(OH)D_3_ deficiency, this may account for one possible mechanism by which VD3 deficiency exacerbates CRSwNP. Both pDCs and cDCs were not statistically increased in mice with dual Af-CRS and VD3 deficiency versus Af-CRS alone. Given that DCs have been shown to generate their own 1,25(OH)_2_D_3_ [61], it is possible that other DC subsets may more efficiently use the low levels of 25(OH)D_3_ found in VD3 deficient mice, and as such are less susceptible to the effects of dietary deficiency.

Next we examined the impact of VD3 deficiency on sinonasal T-cell infiltrates by flow cytometry. While VD3 deficiency did not exacerbate any Af-CRS-induced changes in the percent of sinonasal T-cells present, it alone had a significant impact on the T-cell population present as compared to controls. In fact, the changes in sinonasal CD4+ T-cells and T-regs were nearly identical in mice with Af-CRS as compared to those with only VD3 deficiency. Studies using OVA to induce atopy found that VD3 deficiency did not exacerbate changes in CD4+ T-cells or T-regulatory cells [92], similar to what we have reported here. While we did not find any changes in sinonasal CD8+ cells associated with Af-CRS, we did find that VD3 deficient mice had a significant reduction as compared to controls. The reduction in sinonasal CD8+ cells associated with VD3 deficiency, coupled with reduced IFN-γ, could help to explain the results of human observational studies, which have found low serum 25(OH)D_3_ to be associated with an increased risk of upper respiratory tract infection [93–95].

In addition to immune cell infiltrate, we also examined NALF cytokine levels. In patients with CRSwNP, asthma and cystic fibrosis, VD3 deficiency has been shown to be associated with increased levels of type 2 and Th2 cytokines [27, 96, 97]. Similar to humans with CRSwNP or AFRS, Af-CRS mice displayed Th2 skewed profile that also displayed heightened local levels of the pro-inflammatory cytokines IL-6 and TNF-α [57]. Interestingly, with regard to IFN-γ, VD3 deficiency completely altered the inflammatory response from reduced IFN-γ versus control to having elevated IFN-γ. Similar reports in mice have also shown that VD3 deficiency can lead to increased circulating levels of IFN-γ in mice with OVA-induced allergic airway disease model [92]. These results vary somewhat with studies in humans with CRSwNP and allergic rhinitis, which found low circulating VD3 was associated with lower levels of circulating IFN-γ [25]. However, our study examined local and not systemic IFN-γ concentrations. We also found that in the upper airway, TNF-α levels were elevated in Af-CRS mice with VD3 deficiency. These elevations in TNF-α are consistent with the elevations in sinonasal macrophages, though the source of TNF-α was not examined herein. House dust mite models of allergic inflammation and murine *A*. *fumigatus* infection models also found that VD3 deficiency increases airway levels of TNF-α [98, 99].

Similar, to the study by Nguyen and colleagues [100], we found that IFN-γ was higher in mice with VD3 deficiency as compared to those that are VD3 replete. However, we observed that while Af-CRS was associated with increased IL-4 levels, VD3 deficiency did not exacerbate changes in Af-induced elevation of IL-4. These differences may be in part due to differences in the samples analyzed. In our studies we utilized freshly isolated NALF which would measure secreted cytokines that have spilled into the nasal mucus, whereas the study by Nguyen and colleagues [100] examined whole lung lysates which would likely measure secreted and stored cytokines. Differences in the type of allergen challenge were also present with freshly isolated Af conidia used in the lower airway and a 50:50 mixture of mycelial extract and culture filtrate extract utilized in the presented studies. Lastly, and perhaps most significantly the differences could be in part due to variations of the immune composition of the upper versus lower airway, and as such further studies are warranted to investigate locational differences in VD metabolic responses.

These studies were not without limitations. One such limitation is that this is a model of atopic CRS, and in humans with CRSwNP, both atopic and non-atopic forms exist. The Af-CRS mouse model most closely resembles AFRS, a subset of CRSwNP with more severe disease and allergies to fungi. Previously we have shown that there is no significant difference in the local 1α-hydroxylase or 1,25(OH)_2_D_3_ levels between patients with non-atopic CRSwNP, atopic CRSwNP or AFRS [35, 36]. As such we believe that the findings from our murine studies would apply to AFRS as well as atopic and non-atopic CRSwNP. While many of the mediators examined were not exacerbated by VD3 deficiency, it may be due to the duration of the model. Models of Af-CRS range from four to twelve weeks of intranasal inoculation. Therefore, it may be possible in a longer model that VD3 deficiency may play a greater role in the exacerbation of disease. An additional limitation of this study is that we only used female mice, particularly given prior reports have shown that gender plays an important role in responses to VD3 deficiency and supplement [98]. Also not addressed in these studies is the potential impact of VD3 deficiency on airway microbiome which could be influencing sinonasal immunity and vice versa. While several reports have suggested a role of VD3 in the regulation of airway microbiome [101, 102], our understanding in this area is still limited. Gaining a great understanding of the multi-faceted and complex roles of VD3 is critical to determining ways to better utilize its anti-inflammatory and anti-microbial properties for the treatment of respiratory diseases.

## Conclusions

Dietary VD3 deficiency results in sinonasal changes in the immunological profile, which in many ways mirror the changes in local immunity observed in mice with atopic airway inflammation. Furthermore, the inflammation associated with Af-CRS is capable of suppressing sinonasal VD3 metabolism causing reductions in local levels of the active VD3 metabolite, 1,25(OH)_2_D_3_. Lastly, dietary VD3 deficiency selectively exacerbates immunological changes associated with Af-CRS.

## Supporting information

S1 File NC3Rs ARRIVE guidelines checklist. (PDF)

S2 File **Table A. Comparison of control and vitamin D deficient mouse food.; Figure A Methods summary for the collection of mouse sinonasal mucosa.** A scalpel was used to make an incision along the frontonasal suture and sagittal suture to expose the nasal cavity. A cerumen hook and fine forceps was used to remove the sinonasal mucosa; **Figure B. Representative dot plot of DC staining in mouse sinonasal tissue.** Dead cells were excluded via 7AAD staining prior to analysis. Cells were identified as either CD11b positive or negative by CD11b (left side panels). Cells in their respective CD11b positive or negative gates were then examined for double positive expression (right side panels), indicated in the blue box in the upper right hand quadrant. SSC = side scatter.; **Figure C. Representative dot plot of T-cell staining in mouse sinonasal tissue.** Dead cells were excluded via 7AAD staining prior to analysis. T-cells were identified as either (**A**) CD4+ or (**B**) CD8^+^. (**C**) To identify T-regulatory cells, CD4+ T-cells were subjected to additional gating to identify cells that were CD4+CD25+FoxP3+. T-regulatory cells accounted for <1% of viable cells in mouse sinonasal tissue. Positive gating indicated by blue boxes. SSC = side scatter.; **Figure D. Dietary VD3 deficiency does not significantly alter disease associated changes in serum total IgE concentrations.** Total IgE was measured by ELISA. n = 12–18 mice/group. *p<0.0001 between indicated groups.
(DOCX)

## Abbreviations

1,25(OH)_2_D_3_: 1α,25-dihydroxyvitamin D_3_
25(OH)D_3_: 25-hydroxyvitamin D_3_
Af: *Aspergillus fumigatus*
Af-CRS: *Aspergillus fumigatus-*induced chronic sinusitis
AFRS: Allergic fungal rhinosinusitis
BCA: bicinchoninic acid assay
CBA: cytometric bead array
cDC: conventional dendritic cell
CRS: chronic rhinosinusitis
CRSwNP: chronic rhinosinusitis with nasal polyps
DC: dendritic cell
EIA: enzyme immunoassay
ELISA: enzyme-linked immunosorbent assay
EpCAM: epithelial cell adhesion molecule
FACS: fluorescence-activated cell sorting
IFN-γ: Interferon-γ
IL: Interleukin
MFI: mean fluorescence intensity
moDC: monocyte-derived dendritic cell
NALF: nasal airway lavage fluid
OVA: ovalbumin
pDC: plasmacytoid dendritic cell
TNF-α: tumor necrosis factor-α
T-reg: T-regulatory cell
VD3: vitamin D3 (cholecalciferol)
VD3(-): VD3 deficient
VDR: vitamin D receptor
