# Supplementary material for: Dietary vitamin D3 deficiency exacerbates sinonasal inflammation and alters local 25(OH)D3 metabolism
Source: PLoS One. 2017 Oct 18;12(10):e0186374. doi: 10.1371/journal.pone.0186374 (PMC5646812; doi:10.1371/journal.pone.0186374)
Supplement: S2 File — Table A. Comparison of control and vitamin D deficient mouse food.; Figure A Methods summary for the collection of mouse sinonasal mucosa. A scalpel was used to make an incision along the frontonasal suture and sagittal suture to expose the nasal cavity. A cerumen hook and fine forceps was used to remove the sinonasal mucosa; Figure B. Representative dot plot of DC staining in mouse sinonasal tissue. Dead cells were excluded via 7AAD staining prior to analysis. Cells were identified as either CD11b positive or negative by CD11b (left side panels). Cells in their respective CD11b positive or negative gates were then examined for double positive expression (right side panels), indicated in the blue box in the upper right hand quadrant. SSC = side scatter.; Figure C. Representative dot plot of T-cell staining in mouse sinonasal tissue. Dead cells were excluded via 7AAD staining prior to analysis. T-cells were identified as either (A) CD4+ or (B) CD8+. (C) To identify T-regulatory cells, CD4+ T-cells were subjected to additional gating to identify cells that were CD4+CD25+FoxP3+. T-regulatory cells accounted for <1% of viable cells in mouse sinonasal tissue. Positive gating indicated by blue boxes. SSC = side scatter.; Figure D. Dietary VD3 deficiency does not significantly alter disease associated changes in serum total IgE concentrations. Total IgE was measured by ELISA. n = 12–18 mice/group. *p<0.0001 between indicated groups. (DOCX) [file pone.0186374.s002.docx]

| S1 Table : Comparison of control and vitamin D deficient mouse food | | |
| --- | --- | --- |
|  | **Formula (g/Kg)** | |
|  | **Control Diet**  **(TD 89124)** | **VD3 Deficient Diet**  **(TD 89123)** |
| Vitamin D3 | 2.2 IU/g | 0 |
| Casein, "Vitamin-Free" Test | 180 | 180 |
| L-Cystine | 2 | 2 |
| Dextrose, monohydrate | 644.18 | 644.18 |
| Corn Oil | 100 | 100 |
| Cellulose | 30 | 30 |
| Mineral Mix, Ca-P Deficient | 13.37 | 13.37 |
| Calcium Carbonate | 11.63 | 11.63 |
| Potassium Phosphate, dibasic | 4.9 | 4.9 |
| Potassium Phosphate, monobasic | 3.9 | 3.9 |
| Corn Starch | 1.2214 | 1.2214 |
| Choline Dihydrogen Citrate | 3.497 | 3.497 |
| Vitamin E, DL-alpha tocopheryl acetate (500 IU/g) | 0.242 | 0.242 |
| Vitamin A Palmitate (500,000 IU/g) | 0.0396 | 0.0396 |
| Vitamin Mix, w/o choline, A, D, E | 5.0 | 5.0 |
| Ethoxyquin, antioxidant | 0.02 | 0.02 |


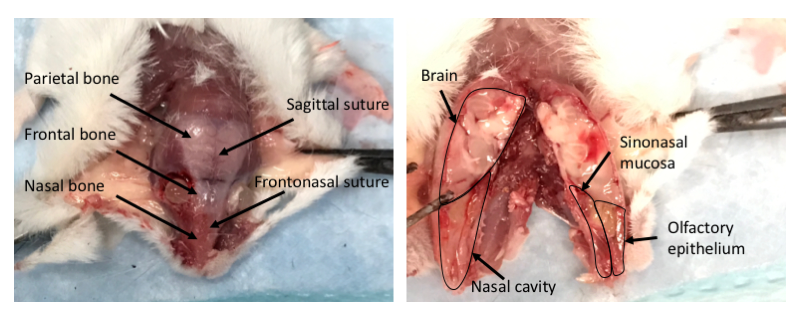


**S1 Fig: Methods summary for the collection of mouse sinonasal mucosa.** A scalpel was used to make an incision along the frontonasal suture and sagittal suture to expose the nasal cavity. A cerumen hook and fine forceps was used to remove the sinonasal mucosa.

**Supplemental Figure 2:**

**
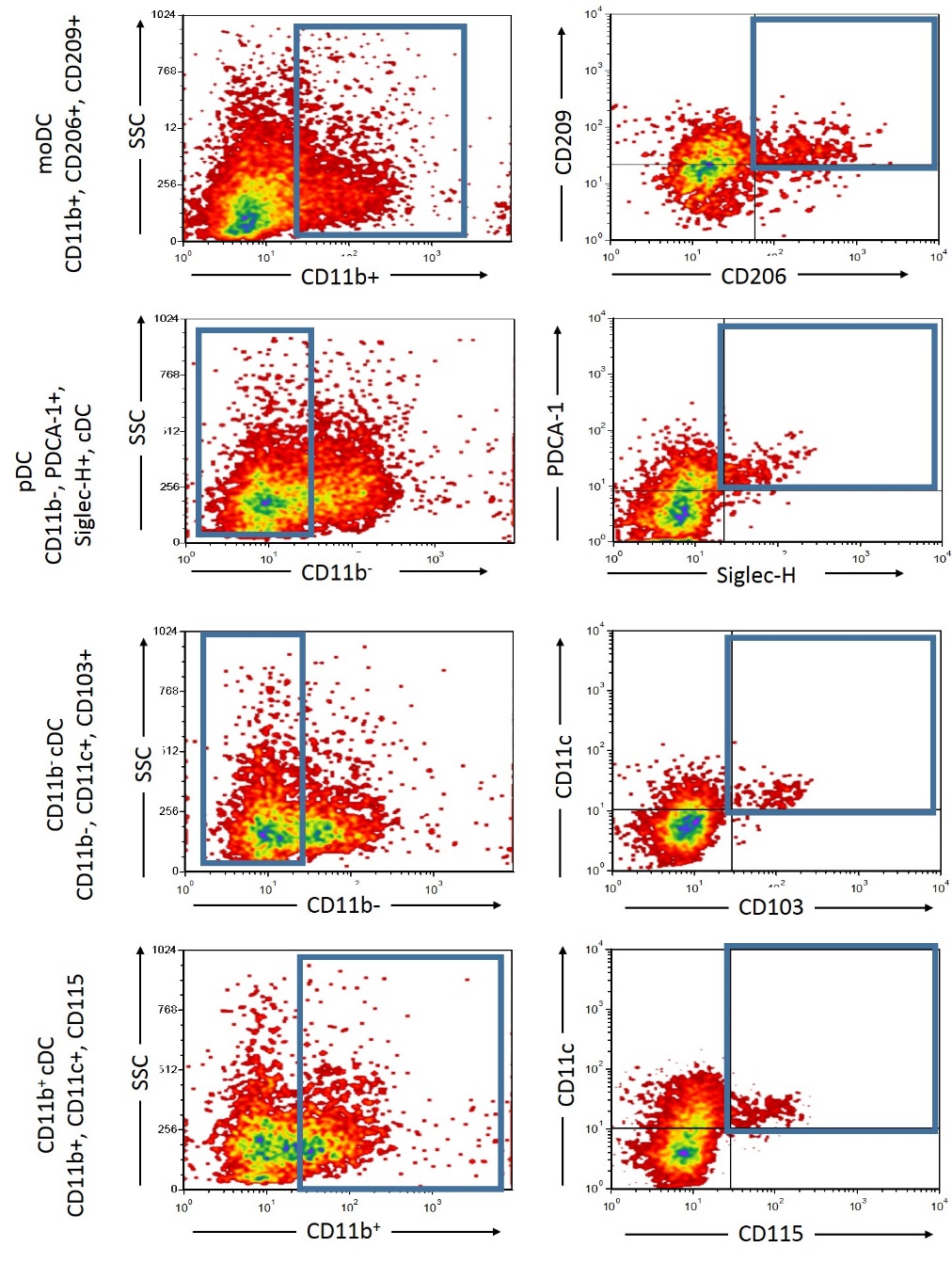
**

**S2 Fig: Representative dot plot of DC staining in mouse sinonasal tissue.** Dead cells were excluded via 7AAD staining prior to analysis. Cells were identified as either CD11b positive or negative by CD11b (left side panels). Cells in their respective CD11b positive or negative gates were then examined for double positive expression (right side panels), indicated in the blue box in the upper right hand quadrant. SSC = side scatter.

**Supplemental Figure 3:**

**
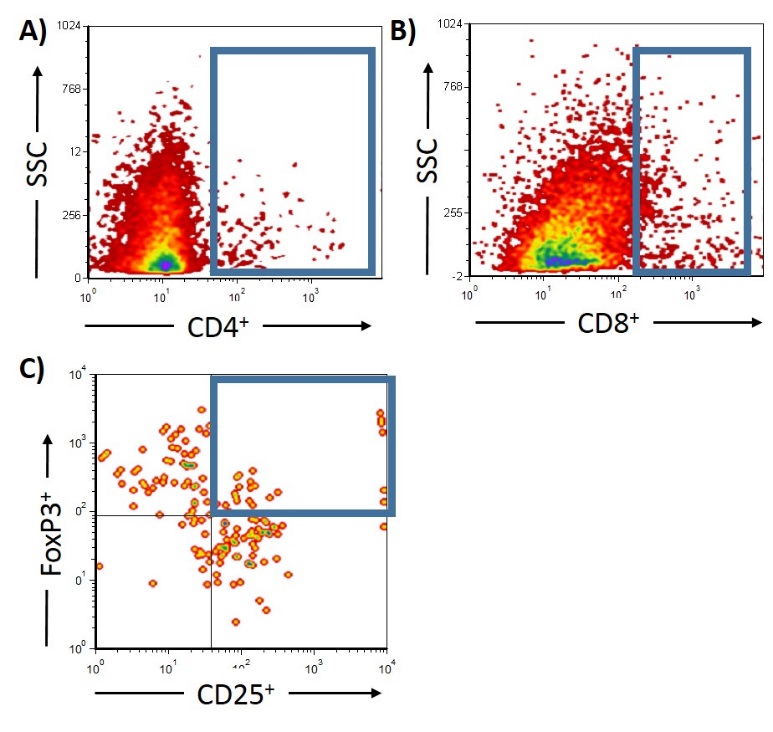
**

**S3 Fig: Representative dot plot of T-cell staining in mouse sinonasal tissue.** Dead cells were excluded via 7AAD staining prior to analysis. T-cells were identified as either (**A**) CD4+ or (**B**) CD8^+^. (**C**) To identify T-regulatory cells, CD4+ T-cells were subjected to additional gating to identify cells that were CD4+CD25+FoxP3+. T-regulatory cells accounted for <1% of viable cells in mouse sinonasal tissue. Positive gating indicated by blue boxes. SSC = side scatter.

**Supplemental Figure 4:**


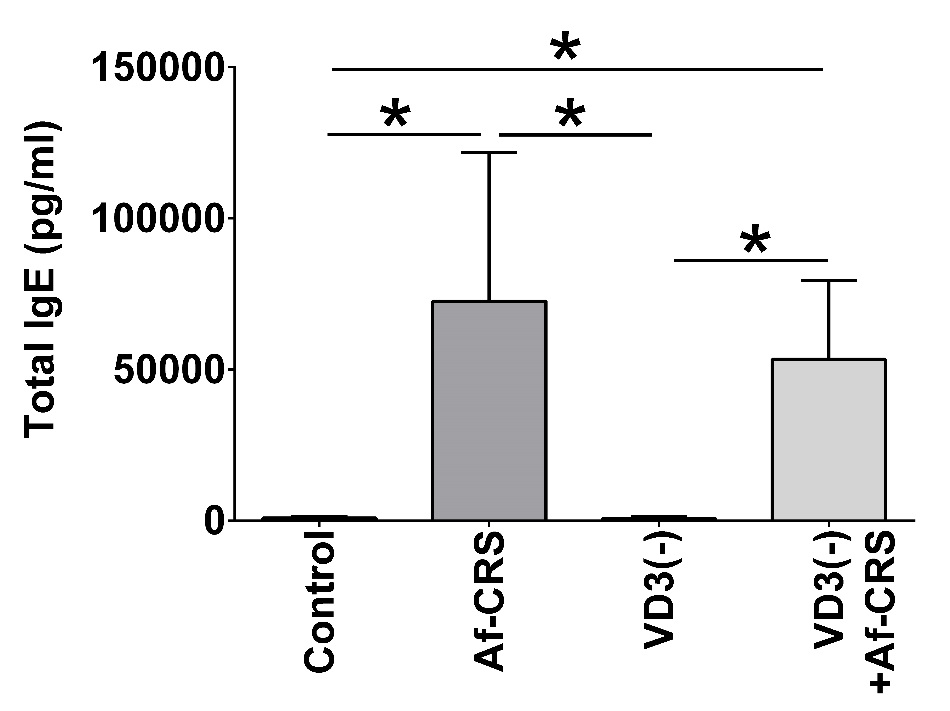


**S4 Fig: Dietary VD3 deficiency does not significantly alter disease associated changes in serum total IgE concentrations*.*** Total IgE was measured by ELISA. n=12-18 mice/group. *p<0.0001 between indicated groups.
